# Critical reviews of immunotheranostics

**DOI:** 10.7150/thno.48966

**Published:** 2020-06-11

**Authors:** Xiaoyuan Chen, Mingnan Chen

**Affiliations:** 1 Laboratory of Molecular Imaging and Nanomedicine, National Institute of Biomedical Imaging and Bioengineering, National Institutes of Health, Maryland 20892, USA; 2 Department of Pharmaceutics and Pharmaceutical Chemistry, University of Utah, Salt Lake City, Utah 84112, USA

**Keywords:** Immunotherapy, theranostics, drug, imaging, screening

## Abstract

Immunity is the most critical and well-regulated protection to the body. Immunity is implicated in a wide range of diseases and serves as the foundation for immunotherapy. Immunotheranostics is the idea of improving immunotherapy through the organic integration of therapeutic, diagnostic, and screening technologies. This special issue collects reviews and opinions from prominent contributors in the immunotheranostic field who represent highly diversified research expertise. The immunotherapeutics discussed in this issue range from small molecules, peptides, antibodies, nanoparticles, and to cells. Discussions from the therapeutic development perspective are accompanied by opinions from the biology and medicine aspects. Further, there are reviews about different types of imaging technologies and their applications in immunotherapy. Lastly, one review raises attention to mass spectrometry for its utilization in the diagnosis and assessment for immunotherapy. In summary, this special issue is a showcase for what is happening in immunotheranostics. Moreover, it is also a justified wish list for what should and will happen in immunotheranostics.

## Introduction

Immunity is the ability of the body to prevent or avoid harm from infections and endogenous disorders. The ability is sustained by the complex immune system consisting of multiple organs and many types of effector cells and molecules. To protect the body, the immune system has to constantly and precisely execute four main functions: immune recognition, immune effector activity, immune regulation, and immune memory. The normally fine-tuned and robust immune system, however, may sometimes be overwhelmed by novel infections such as the SARS-CoV-2 virus 1 or endogenous disorders like malignancy. On other occasions, disorders occur to the immune system itself, which is exemplified by immune deficiency and autoimmune diseases. There are also cases where immunity has to be re-tuned to successfully implement medical treatments, such as suppressing immune rejection for organ transplantation. Collectively, the immune system and immunity are implicated in various types of diseases and medical conditions. Understandably, many medical treatments rely on the modulation of immunity. These treatments, generalized under the term immunotherapy, have a long history. For example, vaccination, a common type of immunotherapy, can be dated back to the eighteenth century. Still, in the last several years, immunotherapy scored a series of breakthroughs, and its research interest and efforts grew at an accelerated pace. Research on cancer immunotherapy, particularly, grew dramatically after the first cancer immune checkpoint inhibitor, Yervoy, was approved in 2011. Such growth offers an opportunity for theranostics to contribute to and shape immunotherapy.

Theranostics is a term coined to denote research efforts that aim at integrating imaging and therapeutic technologies 2. Theranostics can provide real-time information about pharmacokinetics, pharmacodynamics, and molecular and cellular responses to drugs. These categories of information are essential to guide the development, refinement, and application of immunotherapy. Theranostics not only provides new immunotherapeutic methods but also new diagnostics, screening, and monitoring modalities to evaluate and triage immune disorders, and assess the effectiveness of immunotherapies. Interestingly, both immunotherapy and theranostics underwent significant developments in the last decade. The interest in both fields and the interplay between them remains strong. The interplay, encompassed under the term “immunotheranostics” is manifested as the application of existing theranostic technologies to immunotherapy, as well as the utilization of the knowledge and needs originated from immunotherapy research to guide the growth of theranostics. The enthusiasm for immunotheranostics inspired the theme of this special issue that was planned as a platform to voice opinions on this interplay.

This issue starts with a recounting of therapeutic strategies that are under preclinical and clinical developments. These strategies either directly modulate immunity or complement other immunotherapeutic agents. Many of these therapeutic strategies were designed, applied, and herein reviewed around the immune checkpoint blockade, the most active arm of cancer immunotherapy at this moment. The molecules and materials that enable these strategies range from small molecules reviewed by Chen's and Zhu's groups 3, 4, peptides by Li's group 5, RNA by Shi's group 6, antibodies by Breckpot's and Lu's groups 7, 8, and cells by Mo's team 9. Further, Price's group discusses ultrasound as a modality of immunotherapy 10. Among these strategies and molecules, T cell receptor (TCR)-like antibodies are particularly intriguing for their ability to recognize epitope markers on cell surfaces that are indeed derived from intracellular antigens 7. Since most current antibody therapeutics were developed for cell surfaces or released antigens, TCR-like antibodies, for their distinct ability to target intracellular proteins, will significantly expand the target territory of antibody-based theranostics. Together, the aforementioned molecules and materials serve as either active agents or formulation supplements. In terms of drug formulations and delivery, Zhang's and Kim's groups highlight nanoparticles as an important tool 11, 12. From the cancer immunology perspective, the interplay between theranostic technologies and specific immune pathways are also examined. Such examples include the discussion of cyclic dinucleotide-based stimulators of interferon genes (STING) by Zhu's group 4 and the discussion of nanomaterials for analyzing immunosuppressive and immunostimulatory cells in the tumor microenvironment by Huang's group 13.

This special issue also includes discussions of several diagnostic and screening technologies and their application in immunotherapy. De Vries's review discusses the application of two imaging technologies, single-photon emission computed tomography (SPECT) and positron emission tomography (PET), for selecting patients and evaluating responses to immune checkpoint therapy 14. Warram's review, while also on oncological imaging techniques, narrows down to neurological cancer 15. Particularly, Warram's team argues that new imaging breakthroughs would be needed because conventional imaging technology failed to distinguish real tumor progression and pseudo-progression that was merely immunotherapy-induced inflammation. There are also three reviews on monitoring specific types of immune cells, the major labor force of immunity 16-18. Aarntzen's review summarizes *in vivo* imaging technologies for tracking T cells 16. Both Weissleder's and Daldrup-Link's reviews focus on tumor-associated macrophages. While the review of Weissleder's group discusses screening tools to categorize and track the interconversion between different subtypes of tumor-associated macrophages 18; the discussion of Daldrup-Link's group is around macrophage-directed radiotracers and iron oxide nanoparticles in combination with PET and MRI technologies 17. Last but not least, Wang's review, while bearing the same goal to improve diagnosis, takes a different path than imaging 19. Wang's group notes that the classification of patients and the assessment of immunotherapy outcome could be accomplished via mass spectrometry. The review discusses why and how the quantitative mass spectrometry-based biomarkers, which are high-throughput, quantitative, and multiplexing, can be utilized to improve immunotherapy.

Overall, reviews in this special issue showcase highly diverse immunotheranostic efforts to improve immunotherapy and highlight innovative answers to the challenges revealed in immunotherapy clinics, such as pseudo-progression after immunotherapy 15. Meanwhile, these reviews also collectively reflect two current “regrets” of immunotheranostics: first, immunotheranostic research has overwhelmingly concentrated on cancer immunotherapy leaving many other types of immunotherapies largely ignored; second, the most desired capacity of theranostics, the organic integration of therapy and diagnosis, has not yet been fruitful in the clinic. These deficiencies are understandable given the young age of theranostics and the new developments in immunotherapy. Moreover, these “regrets” indeed justify two goals for future immunotheranostic research and could become two highlights in the next special issue of immunotheranostics.
